# The Multimodal Nature of High-Intensity Functional Training: Potential Applications to Improve Sport Performance

**DOI:** 10.3390/sports7020033

**Published:** 2019-01-29

**Authors:** Joao Henrique Falk Neto, Michael D. Kennedy

**Affiliations:** Athlete Health Lab, Faculty of Kinesiology, Sport, & Recreation, University of Alberta, Edmonton, AB T6G 2R3, Canada; kennedy@ualberta.ca

**Keywords:** athletes, sports performance, aerobic power, anaerobic capacity, strength, power, CrossFit, athletes

## Abstract

Training for sports performance requires the development of multiple fitness components within the same program. In this context, training strategies that have the potential to concomitantly enhance metabolic and musculoskeletal fitness are of great value for athletes and coaches. The purpose of this manuscript is to review the current studies on high-intensity functional training (HIFT) and to assess how HIFT could be utilized in order to improve sport-specific performance. Studies on untrained and recreationally-active participants have led to positive results on aerobic power and anaerobic capacity, and muscular endurance, while results on muscular strength and power are less clear. Still, HIFT sessions can elicit high levels of metabolic stress and resistance training exercises are prescribed with parameters that can lead to improvements in muscular endurance, hypertrophy, strength, and power. As similar training interventions have been shown to be effective in the athletic population, it is possible that HIFT could be a time-efficient training intervention that can positively impact athletes’ performances. While the potential for improvements in fitness and performance with HIFT is promising, there is a clear need for controlled studies that employ this training strategy in athletes in order to assess its effectiveness in this population.

## 1. Introduction

Performance in sports is usually determined by an optimal combination of aerobic and anaerobic metabolism, muscular power and strength, and speed, and agility [1]. Of the many training strategies that are employed with the goal of improving sports performance, resistance training plays a key role for athletes in all sports, particularly because of its role in increasing muscular strength and power [2,3,4]. Similar to resistance training, high-intensity interval training (HIIT) has received a great deal of attention recently as a result of its potential to increase aerobic power and anaerobic capacity, which also leading to improvements in performance [5,6,7]. 

While resistance training and HIIT elicit specific adaptations that could increase athletic performance, a combination of both seems to provide an optimal stimulus for the development of physical fitness when compared to performing either training alone [8,9]. However, common to every sport is the fact that training time is limited, particularly considering how each modality has a highly demanding technical and tactical component that must also be mastered [10]. Thus, finding the most efficient way to develop the physical, technical, and tactical components simultaneously is a challenge that athletes and coaches consistently face [4]. 

Circuit training is often employed when there is a need for concomitant improvements in muscular strength, power, endurance, and aerobic and anaerobic fitness [1]. Reports of the use of circuit training are common in the literature [11,12], and coaches often prescribe it as a time-efficient strategy to impose a significant metabolic stress on athletes, while muscular endurance and strength are also challenged [12]. Recently, Hermassi et al. [2] provided further support to the effectiveness of circuit training programs in high-level athletes by showing significant improvements in muscular strength, power, and throwing velocity in a group of handball players over 10 weeks of the competitive season.

However, some circuit training studies in the literature are prescribed with exercises, intensities, and speed of movement that are not optimal for developing muscle strength and power, and consequently, sports performance [13,14]. Exercises performed using explosive movement patterns and with higher loads for the lower body, and moderate loads for the upper body have been shown to be more beneficial when the goal is to maximize power during circuit training [15]. Thus, it is likely that circuit training programs that employ compound exercises at a range of intensities and with different durations, and that utilize explosive actions can lead to even better results in terms of fitness development. In this context, high intensity functional training (HIFT) could emerge as an effective alternative to improve fitness when compared to traditional circuit training.

High intensity functional training incorporates functional exercises (those that involve whole body, universal motor recruitment patterns, executed in multiple planes of movement) in sessions that are intense, short, and constantly varied, with the potential to stress different body systems in a balanced and integrated manner [16,17,18]. Despite its similarities to high intensity interval training, HIFT is unique in the fact that it utilizes a mix of different exercises of multiple modalities, such as Olympic weightlifting, power training, and body weight exercises, often combined with aerobic training, while HIIT is usually unimodal in nature [17,18,19].

Similar forms of training have been previously reported in the literature under different names [17,20,21] with CrossFit recently emerging as a popular form of HIFT among fitness enthusiasts, where gymnastics, weightlifting, and cardiovascular exercises are performed at a high intensity with little to no rest between these exercises [22,23]. Given the fact that many terms have been used in previous studies to indicate HIFT interventions, a clearer definition of the term has recently been proposed in order to ensure consistency in future research. Feito et al. [17] defined high intensity functional training as “a training style (or program) that incorporates a variety of functional movements, performed at a high intensity (relative to an individual’s ability), designed to improve parameters of general physical fitness (cardiovascular endurance, strength, body composition, flexibility, etc.) and performance (agility, power, speed, and strength)”.

The main benefit of HIFT lies in the fact that it can challenge multiple systems in the body in a single session, with the potential to increase aerobic power and anaerobic capacity, as well as muscular endurance, strength, and power, while positively impacting body composition and work capacity [16,17,19]. Similar interventions have shown positive results in athletes [2], and a recent study demonstrated that HIFT elicited a similar improvement in a 5-km performance in recreational runners with a shorter time commitment [24]. It is possible then, that the multimodal nature of HIFT could provide a time-efficient alternative to traditional resistance and circuit training. Thus, the purpose of this manuscript is to review the current studies on HIFT, and to assess how this training style could be utilized in order to improve sport performance. A brief overview of the key points regarding the use of HIFT to improve fitness are summarized in Table 1. 

## 2. HIFT and Metabolic Adaptations for Sport Performance

The intense nature of HIFT has been highlighted recently. Kliszczewicz et al. [28] compared the cardiovascular response of a training session known as “Cindy” (as many rounds as possible of 5 pull-ups, 10 push-ups, and 15 body weight squats in 20 min) to 20 min of high intensity running at 90% of the participants’ maximum heart rate (HRmax). Both sessions elicited high levels of oxidative and metabolic stress with no significant differences between them. Heart rate and rate of perceived exertion (RPE) were analyzed at 6-, 10-, 16-, and 20-min, with both training protocols showing gradual increases in these measures. However, “Cindy” was rated as being more demanding by participants, as measured by higher RPE scores at all time points, in addition to higher heart rate values, with participants achieving 97% of their maximal heart rate by the end of the session. Corroborating these findings, Fernández-Fernández et al. [27] also showed heart rate values of around 97% of the participants’ maximum for the same training session. The authors analyzed another training session, “Fran”, comprised of barbell front squat and overhead press (i.e., a “thruster”) and pull-up exercises, performed back to back, for three rounds, with participants required to perform 21 repetitions of each movement, then 15 repetitions, then 9 repetitions, as quickly as possible, with no rest time permitted between movements or between rounds. The participants averaged 94% of their maximum heart rate at the end of the session, and despite the small difference in heart rate values between them, both sessions had similar blood lactate values, with an average above 14 mmol immediately following their completion. Other research studies have corroborated such findings and highlight how metabolically demanding a HIFT session can be [19,29].

This level of metabolic stress is the target of many HIIT sessions. When investigating the interaction between interval duration and intensity, Seiler et al. [30] showed that 4- and 8-min intervals, performed at approximately 95% and 90% of participants’ HRmax, respectively, were effective in improving endurance performance in recreational cyclists. Interestingly, these heart rate values were very similar to what was reported at the 6- and 10-min mark during the “Cindy” training session (93.3 ± 1.2% and 94.6 ± 0.9%, respectively) [26]. The HIFT sessions also achieved a higher RPE score and lactate levels when compared with the 4- and 8-min intervals [29], although this might be due to the duration and nature of the sessions. As reports of performance improvements following unimodal HIIT are common in the literature [5], these findings suggest that at least when matched in duration, HIFT sessions can induce a similar stress to a traditional aerobic interval workout [26]. In this context, HIFT could be used as a form of cross-training, providing a viable alternative to sport-specific training methods. 

Considering that high levels of metabolic stress in HIFT sessions were achieved even when only performing resistance training exercises [27,28], sessions that utilize short, intense aerobic intervals could have an even greater impact on the athlete’s aerobic performance. For example, short, intense intervals, lasting from 20 s to 4 min, are common in many HIFT programs [16,29,31,32], with these durations covering a wide range of possible metabolic adaptations that can occur with interval training [5]. These interval durations have been shown to be effective in improving not only aerobic power, but anaerobic capacity as well [7,11,30]. Thus, when planning HIFT sessions, such intervals could be performed earlier in the session, by themselves or as part of a circuit, to provide a further stimulus to athletes, as shown in research. 

This is commonly seen in many HIFT programs with different studies using rowing (250 m for approximately 45 to 60 s) combined with other exercises that would maintain a high level of metabolic stress (burpees, kettlebell swings, and kettlebell thrusters, for example) [29,32]. Crawford et al. [16] and Outlaw et al. [31] also reported the use of short, intense bouts of exercise (400 m to 800 m of running), with these aerobic intervals also prescribed as the first exercise of a circuit. In addition, some forms of HIFT, such as CrossFit, even take advantage of longer bouts of traditional cardiovascular exercises as part of their metabolic sessions, such as 2-mile runs, 5-km rows, and 8-km partner rows [16,33].

Recently, Carnes and Mahoney [24] compared the effects of a multimodal training program that combined interval training with HIFT sessions to a polarized endurance training program on maximal oxygen consumption (VO_2max_) and 5-km performance in a group of recreational runners. Over 12 weeks, the multimodal group performed short (30–60 s) and long intervals (1.5–6 min) at the participants’ maximal sustainable effort, in addition to HIFT sessions consisting of a strength activity followed by an intense circuit, while the polarized training group followed a traditional running program. While the improvements in the VO_2max_ were greater for the polarized group (4.3 ± 3.6 vs. 1.78 ± 1.9 mL·kg·min^−1^), performance in the 5-km time-trial improved to the same extent in both groups, leading the authors to conclude that recreational runners can obtain a similar degree of improvement in the distance using either approach [24]. In addition, the multimodal training group had a lower running volume during the intervention, highlighting the potential that HIFT has as a time-efficient training strategy. 

In addition, as HIFT is varied in nature, with different modalities, exercises, and durations employed in various sessions, it is possible to manipulate the training parameters in order to mimic the specific demands of a sport. Cross country skiers, for example, could perform circuits utilizing activities that require a high number of repetitions targeting the elbow extensors, at a lower percentage of the athletes’ maximal voluntary contraction, therefore enhancing muscular endurance while providing a significant metabolic stimulus which would lead to improvements in the athletes’ cardiovascular system. This is what is seen in Table 2, where a sample training session is described. The utilization of short intervals as the first exercise of a circuit is a common practice in many HIFT sessions [16,29,31,32], and the subsequent exercises work movement patterns and muscles that are commonly targeted in the sport, while still maintaining the high level of metabolic stress that is induced by the first exercise. The outline of the session, with an 8-min interval, is conducive to improvements in VO_2 max_, a key characteristic for performance in endurance sports, with intervals of this duration shown to be effective in eliciting such improvements [30]. This session would allow for athletes to perform a high number of repetitions at a lower percentage of their maximal voluntary contraction with short recovery periods between exercises, thus truly enhancing the specific muscular endurance characteristics that are typical of the sport. 

Similarly, training sessions for sports such as rock climbing, swimming, and wrestling, could involve shorter bouts of intense exercise (2 to 7 min), with specific exercises that can mimic the musculoskeletal demands of each sport, with or without the addition of cardiovascular exercises [12,34,35]. When applied to team sports, sessions could utilize the multi-joint exercises that are common to training programs in these modalities, performed in a continuous circuit, interspersed with short bouts of intense sprints, as commonly occurs in these sports [36,37]. This outline is very similar to what was used to improve handball performance in a group of high-level players [2] with the use of heavy strength exercises (squats and bench presses) combined with plyometrics and short sprints. 

Additionally, by mirroring the specific work to rest ratios experienced during competition, it is likely that the cross-training potential of HIFT could be enhanced. In this context, HIFT sessions performed as AMRAPs (as many rounds as possible) of a few specific exercises (with durations usually ranging from 5 to 20 min) could mimic the demands of power-endurance sports, such as track cycling, rowing, swimming, and canoeing or kayaking [38], or combat sports, such as judo or mixed-martial arts [39,40]. An example of how this can be used to develop aerobic power in martial arts athletes is provided in Table 3. This training session is adapted from the CrossFit session “Fight Gone Bad”, developed to simulate the high level of effort that is required in martial arts by mimicking the work to rest ratios that occur in these sports. However, the original session utilizes exercises aimed at developing muscular power, such as sumo deadlift high pulls, push presses, and box jumps, with higher repetitions. The program has been modified to an extent to bring its parameters closer to what is currently known as best practices in the field when it comes to the use of such exercises [41,42]. Thus, the exercises were adapted from Kostidiakis et al. [13] to still reflect the muscular demands faced by athletes. As the aim is to improve aerobic power, the 5-min intervals would lead to a significant metabolic stimulus, with Tibana et al. [18] reporting HR values above 90% of the participants’ maximum, and lactate concentrations of 17.2 mmol with the original training session. In addition, the performance of three to five rounds, with only one to two minutes of rest between rounds is similar to the work to rest ratios that have been shown to be effective in improving VO_2max_ in HIIT studies [5,30].

Similarly, “rounds for time” where participants are encouraged to complete the total work as fast as possible can be used to create sessions that are comparable to shorter intervals (four to eight minutes) or that resemble a longer exercise bout, with coaches manipulating the duration of the session by altering the number of exercises and repetitions performed [16,31]. This format could mimic shorter, intense events such as short-track speed skating or team sprints in Nordic skiing, or longer events similar to what has been previously mentioned with AMRAPs. Intermittent sports can benefit from the use of circuits with timed-sets (usually 30 to 90 s for each station), to mimic specific work to rest ratios in sports such as soccer, handball, and field hockey, or shift duration in ice hockey [16,18,19,21]. Although this concept of how HIFT can be designed to elicit the demands of specific sports has not been researched, it has been applied in occupational settings so as to enhance tactical activities such as firefighting [26].

In addition to the possible benefits to the aerobic and anaerobic systems, HIFT sessions could also improve what has been termed “general athletic ability” [16], leading to an increased work capacity. Indeed, these improvements represent the largest reported effects of HIFT interventions to date [33], and are a result of the functional tasks that are part of the programs challenging the integration and efficiency of many body systems at once [17]. The improvements in general athletic ability and work capacity, particularly in the off-season, could increase the athletes’ general fitness to the point where they may be able to tolerate higher sport-specific training loads during the pre-season, possibly leading to better results overall.

## 3. HIFT to Improve Muscular Strength, Power, and Endurance

While necessary for many sports, the performance of endurance and resistance training as part of a training program or within the same training session has been termed concurrent training [43]. When these components are trained together, it is believed that an interference effect would occur and adaptations to each training mode would be limited [44]. However, once thought to be a key limitation, such an interference effect has been the target of much debate recently, with research providing mixed results [9,45,46]. This is likely due to the fact that many factors (including exercise volume, intensity, and nutritional state, among others) contribute to the occurrence of the interference effect, with recent evidence showing that such an interference effect might also depend on the overall fitness level of the participants, their training experience, and the frequency of sessions in a week [44]. 

Particularly, when resistance and endurance sessions are performed at separate times during the day, such an interference effect does not seem to influence the athletes’ responses [8,9]. Supporting this hypothesis, Fyfe et al. [47] recently demonstrated that even when combined within a short period (15 min), an acute bout of high intensity interval training and heavy resistance exercise did not compromise the signaling cascade associated with muscular adaptations. Still, when performed in the same session, the order of exercises seems to be important, with research indicating that if maximizing strength is the goal, strength exercises should be performed prior to endurance training [48,49]. As evidence seems to indicate that athletes who do not specialize in pure endurance or strength disciplines can have concomitant improvements in muscular strength and power and endurance performance, it is possible that an interference effect might not be an issue when HIFT is applied to many sports. 

In fact, it has been shown that the high number of repetitions performed and the intense nature of weightlifting exercises in HIFT sessions have the potential to increase muscle endurance, hypertrophy, strength, and power [2,21,23,25]. De Sousa et al. [23] examined the differences in fitness between recreational CrossFit participants and resistance-trained individuals who were required to have at least one year of training experience and a current training frequency of at least two to three sessions per week. According to the authors, both groups outperformed the average results for male individuals of a similar age in a pull-up test, while also achieving counter-movement jump scores similar to that of age-matched soccer players, with the results slightly favoring the CrossFit group. Although the authors recommend caution when interpreting these results given the cross-sectional design of the study, it is suggested that HIFT training could be as effective as traditional resistance training for improving muscular strength, power, and endurance [2,21,23,25]. A training intervention in college students compared the effectiveness of a CrossFit program to a traditional training program and showed that the CrossFit group had the largest improvements in lower body muscular endurance [25]. Although the CrossFit program was not as effective in improving muscular strength and power as the traditional training group, 60% of the participants improved their lower body power, with approximately half of then showing improvements in upper body strength [25].

Corroborating these findings, Heinrich et al. [21] demonstrated that a HIFT program was more effective in eliciting improvements in muscular endurance than the traditional army training program. Over a period of eight weeks, the authors had the participants in the HIFT group complete high intensity circuits consisting of 15 exercises focused on strength, power, speed, and agility. The exercises included Olympic lifts, plyometrics, and upper and lower body exercises, in addition to core exercises, with each exercise performed for 60 to 90 s, while little to no rest was allowed between the stations [21]. Contrary to what had been previously reported [25], the high-intensity functional training group had significantly larger improvements in upper body strength, as measured by a bench press 1RM test [21]. While not statistically significant, similar to the findings of de Sousa et al. [23], the HIFT program also showed better results in vertical jump performance when compared to the traditional army training group [21]. 

Research on high intensity resistance circuit training (HICT) has also demonstrated positive results. HICT differs from HIFT, as it is often prescribed at a single intensity (usually six to eight RM), with circuits having a very short recovery between exercises and a longer recovery between sets, while sometimes employing single-joint exercises, thus not matching the constant variance across exercises, intensities, and time-domains that are typical of HIFT. Still, Hermassi et al. [2] showed that a circuit resistance training program could be used to increase fitness and sport-specific characteristics in a group of top-level handball players. The participants were divided into an experimental group who replaced two sessions (a strength with moderate loads, 55–70% 1RM and a technical and tactical session) with the circuit training program, and a control group who maintained their training routine. The circuit training program focused on strength and power, with activities such as jumping and sprinting combined with resistance training that lasted on average 35 min. After 10 weeks of training, the experimental group demonstrated greater improvements in muscle volume, strength, and jumping ability [2]. The control group, meanwhile, not only had no significant improvements in the tested variables, but had a reduction in several of them [2]. This highlights what can be a common problem in team sports, where the lack of training time during a long competitive season may lead to a reduction in the fitness components that are related to sports performance [50]. Considering that the improvements occurred with short training sessions [2], it is possible that HIFT can be used as a time-efficient strategy to maintain or improve fitness during the competitive season. 

The improvements in muscular endurance following HIFT, in particular, are not surprising. Many sessions involve the completion of a high number of repetitions of multiple exercises in short periods of time [14,16,21], characteristics that are typical of muscular endurance programs [51]. Traditional sessions used in CrossFit, such as “Cindy”, “Fran”, and “fight gone bad”, and other circuits employed in research studies provide clear examples [16,27,29,32]. In addition, the use of longer timed-sets (30 to 90 s), as used by Heinrich et al. [21], is common in HIFT programs and is also reflective of muscular endurance programs. 

Similar to what occurs with muscular endurance, sets and repetition ranges that resemble those traditionally prescribed for muscular hypertrophy [51] are very common in HIFT sessions, with this occurring particularly in the sessions that combine gymnastics and weightlifting exercises [16,19,31]. In these sessions, exercises are often performed to the point of muscular failure, which has been shown to be effective in eliciting gains in muscle mass at different repetition ranges [52]. While not an emphasis of many athletic programs, muscular hypertrophy still is considered an important component to be developed [53], particularly earlier in the off-season, as part of general fitness development. 

In addition, specific weightlifting sessions are common in HIFT, particularly in CrossFit. Crawford et al. [16] reported that in a full month of training, with five sessions per week, weightlifting exercises were performed at least two to three times per week, and sessions were often devoted exclusively to this component. In such sessions, participants gradually work their way to near-maximal or maximal lifts, usually in exercises that are commonly performed in athletic settings, such as squats, deadlifts, and Olympic lifts. As the prescription of the specific parameters are very indicative of traditional strength training (one to five reps, three to eight sets, 85% to 100% 1RM) [51], this supports the few studies to date that have highlighted the potential that HIFT has in increasing participants’ muscular strength [2,21,23]. 

Considering that increases in strength would have a direct transfer to the development of muscular power [41], previous studies have demonstrated that HIFT has the potential to increase this characteristic as well [2,21,23]. However, as some of the positive results have not reached statistical significance [21,23], it is likely that the development of power is one area where common HIFT prescriptions could be modified to provide further benefits for athletes. Many HIFT sessions commonly use high repetitions of explosive exercises, such as Olympic lifts and plyometrics [16,29], practices that are currently not supported by evidence. Training for muscular power should follow traditional guidelines (one to five reps, and three to six sets) with exercises performed at different intensities focused either on high velocity (30–60% 1RM) or high force development (>80% 1RM) [42,51]. Thus, an approach that takes advantage of training at different ranges of the force x velocity curve (heavy resistance training, ballistic and explosive resistance training, or a mixed resistance training response) would lead to improvements in the rate of force development at each point of the curve, and is likely to provide the greatest gains in power development [41]. However, when such exercises are performed for high repetitions, neither one of these aspects would be satisfied and it is possible that improvements would not occur. In addition, Booth and Ohr [54] report that in plyometric training, the optimal number of foot contacts per session would range between 30 and 80, varying according to the sport and time of the year, in addition to long rest periods between sets, which is different than what some HIFT sessions reported in the literature [16].

Thus, when applied to HIFT, exercises such as Olympic lifts and plyometrics, could be performed early in the training session, apart from circuits, using sets, repetitions, and rest periods, which are conducive to the development of muscular power [41,42,51]. This is what was reported by Tibana et al. [32], with participants performing snatches and clean and jerks with low repetitions, and two to five minutes of rest between sets. An intense circuit only took place following the completion of these exercises and a 5-min recovery. Still, it is possible that power-oriented exercises could also lead to positive results when performed as part of a short circuit designed to keep metabolic stress low and mechanical stimulus high. This outline was effectively used in handball athletes, where one plyometric exercise was performed by itself early in the session, followed by a circuit that involved heavy resistance training, short sprints, and other plyometric exercises, leading to improvements in vertical jump and sprinting performance [2]. In addition, HIFT could benefit from concepts that have been explored with complex training, a training strategy where heavy resistance training exercises are paired with plyometric or power exercises (weightlifting movements such as the snatch, clean and jerk, and their derivatives), possibly leading to greater gains in muscular power. In a recent meta-analysis [55], it was demonstrated that complex training had moderate to large effect sizes in terms of improving sprint performance and vertical jump, depending on the duration of the program and the characteristics of the athletes. 

A sample training session to enhance muscular strength and power is presented in Table 4. The design of this session, with one power exercise performed by itself early in the training session, has been reported by Tibana et al. [32] and is often commonly used in HIFT sessions when strength and power development are the goal of the session. The combination of back squats and box jumps takes advantage of the concept of complex training, which has been shown to be effective in producing improvements in muscular power [55]. The short circuit involving functional exercises and short sprints is similar to what has been employed by Hermassi et al. [2] to improve sports specific performance in a group of high-level handball players. Lastly, the use of heavy and high velocity exercises ensures the athlete is performing at different points of the force x velocity curve [41], while the number of total foot contacts of the session also follows optimal guidelines [54].

The available evidence demonstrates that HIFT can be an effective strategy in improving muscular endurance, power, and strength, and that sessions can be adapted to target these specific fitness components. Thus, strength and conditioning coaches and practitioners could plan their sessions with specific weightlifting and gymnastics exercises for a set number of repetitions (1 to 15+) using either timed circuits or AMRAPs, depending on the level of metabolic stress that is desired [2,16,21,23]. When specific gains in strength are the goal, sessions would benefit from a focus on weightlifting exercises, with these sessions either performed with a single focus or combined with gymnastics and short metabolic components [16,21]. Still, when performed in the same session, strength exercises should precede endurance ones, if the goal is strength development [48]. Lastly, the development of muscular power could follow a similar outline with a greater focus on specific exercises earlier in the session, either performed by themselves or combined with heavy resistance training, and perhaps even short sprints and plyometrics [2,32,41]. As in most resistance training recommendations for athletes, it seems that two to three sessions per week with a higher volume performed during the off-season should suffice to stimulate the desired improvements in performance. During the competitive season, it might be that one session a week could be enough to maintain the training adaptations that occurred at previous phases of the training plan. 

## 4. Limitations and Considerations When Applying HIFT to Athletes

It is undeniable that HIFT elicits a high level of metabolic stress on participants [18,28,29]. Such stress is as high as what has been reported in single aerobic bouts [28], and is equivalent to what is seen in sessions of HIIT [30]. In addition, sessions that have a greater focus on resistance training exercises are performed in ranges and intensities that are appropriate for the development of muscular endurance, hypertrophy, strength, and power [3,16,21,51]. Thus, if training variables are manipulated accordingly, HIFT should lead to the desired improvements in VO_2max_, anaerobic capacity, and musculoskeletal fitness, which could lead to an improved performance. However, a recent narrative review reported that this might not be the case, with HIFT having little to no effect on the improvements in physical fitness [56]. In addition, a meta-analysis [22] reported that studies on HIFT often have a high risk of bias and a low level of evidence, and that an improvement in the quality of studies is necessary so that HIFT can be critically evaluated. 

In light of these findings, the design of some training sessions and programs could partially explain these results. Many HIFT programs, particularly in CrossFit, appear to have too much variability on a daily basis, not allowing for a proper training overload to occur, which would be detrimental to the optimal development of fitness components [16,25,33]. Therefore, when designed to improve performance in athletes, HIFT should be prescribed to allow for consistent progressive overload and training adaptations. Previous research using circuit training and HIIT, with consistent progressions in training intensity or volume, provide further evidence that with proper planning, improvements should occur with these training modalities [2,13]. In addition, session RPE has recently been validated as a tool to monitor internal training loads in HIFT sessions [18], and thus, could be used to monitor training stimulus, allowing for a proper overload.

Another possible explanation for the reported lack of improvements in physical fitness following HIFT programs [56] is the fact that these programs induce a high level of fatigue [19,28,29,33]. Indeed, HIFT programs have been associated with early signs of overreaching, such as negative mood states and higher levels of inflammatory markers in as little as four weeks [33]. In addition, the type of session and exercises used have been shown to elicit different levels of fatigue, with gymnastics and weightlifting exercises having a higher level of neuromuscular fatigue that can take up to 24 to 48 h to dissipate [29]. As many HIFT programs involve three to five consecutive days of training per week [16,33], it is possible that participants are not fully recovered from the training intervention during their post-intervention testing. Thus, coaches and practitioners should consider the high level of fatigue that will be experienced by athletes when planning HIFT programs, allowing for enough recovery to occur between sessions. Still, despite the intensity of the sessions and the high level of fatigue raising concerns over safety, Tibana and Souza [56] reported that the injury rate of HIFT participants is similar to what is seen in weightlifting and most recreational sports, establishing HIFT as a safe training strategy.

Thus, despite the positive effects reported in untrained and recreationally-trained individuals [21,23,25,33], and the high level of mechanical and metabolic stress that HIFT sessions can elicit [19,28,29], caution must be taken when these findings are transferred to an athletic population. While many studies have shown that training sessions with similar levels of metabolic stress are effective in improving aerobic power or anaerobic capacity [30], research has also shown mixed results in terms of fitness improvements in athletes with interventions that have previously been shown to be effective [57]. Still, promising results have been demonstrated in the literature. 

For example, de Sousa et al. [23] have shown that male individuals that had at least one year of CrossFit experience had average VO_2max_ scores of 52.45 mL·kg·min^−1^, values that are similar or above what is seen in male mixed-martial arts athletes [39], and recreational endurance athletes [24,30], to cite a few sports. In addition, the authors [23] have also shown that the CrossFit participants had vertical jump values that were similar to that of age-matched soccer players, highlighting the potential that HIFT has to lead to significant improvements in characteristics that are key to performance in many sports. Similarly, using circuit training during the competitive season, Hermassi et al. [2] were able to achieve significant improvements in sprint performance and vertical jump in a group of high-level handball athletes. Recently, a multimodal training program was shown to be equally effective in improving 5-km time trial performance in a group of recreational runners by using a combination of short and long intervals and HIFT, while having a much lower running training volume [24]. While these results provide evidence that HIFT could have a positive impact when it comes to improving fitness in athletes, the authors still recommend caution in interpreting these results. Future studies that assess the effectiveness of specific HIFT interventions in athletic populations are necessary in order to determine whether this can be a viable training strategy for athletes of different levels and sports.

## 5. Conclusions

High intensity functional training involves the use of functional exercises (those that involve whole body, universal motor recruitment patterns, executed in multiple planes of movement), often combined with intense cardiovascular activities, that have the potential to stimulate different systems in the body in a balanced and integrated manner [16,33]. Current research has shown that HIFT has the potential to lead to improvements in muscular strength, power, hypertrophy and endurance, aerobic and anaerobic performance, body composition, and work capacity in untrained and recreationally-trained participants [2,21,23,24]. Still, considering the high level of metabolic stress that is elicited by HIFT sessions [27,28,29], how the prescription of resistance training exercises can be indicative of traditional muscular strength and power training [16,42,51] and how circuit training has been shown to be effective in improving fitness in athletes [2,24], it is proposed that HIFT could be a time-efficient strategy particularly for those sports that require the simultaneous development of multiple fitness components for performance.

In this context, athletes from different sports could benefit from adding HIFT to their training programs, particularly during the off-season and pre-season. The performance of HIFT during the competitive season could also be a time-efficient strategy to maintain, and even increase, specific sports performance [2]. When the focus is on improving aerobic and anaerobic fitness, athletes could perform sessions that include any combination of metabolic, weightlifting, and gymnastics exercises, with sessions performed as AMRAP (as many rounds as possible), where recovery is minimal or non-existent, or with a set number of repetitions completed as fast as possible, providing an effective way to elicit high levels of metabolic stress [27]. In addition, coaches can plan sessions to elicit the specific work to rest ratios experienced by the athletes in their sports while using exercises that mimic the musculoskeletal demands of each modality, thus enhancing the cross-training potential of HIFT.

Training parameters can also be manipulated so as to better stimulate muscular adaptations. Muscular endurance seems to be the component that could benefit the most, as many HIFT sessions are performed with short recovery periods and a higher number of repetitions [21,25]. To optimize muscle strength, it is recommended that weightlifting exercises be the focus of the training session, combined or not with other exercises in short circuits that might involve explosive exercises. The development of muscular power should follow similar guidelines, and it must be acknowledged that higher repetitions of explosive exercises, although common in some HIFT settings, might not provide an optimal stimulus to the development of this quality [41,42] and should be avoided. Lastly, coaches should understand that HIFT can generate significant levels of fatigue [19,28,29,33] and a high overall training load. Therefore, sessions should be planned according to the goals of the specific training phase, and enough recovery should be provided between sessions. In addition, while a high variety of exercises and sessions might be attractive to the general population, when designed to improve performance in athletes, HIFT should be prescribed to allow for consistent progressive overload and training adaptations, with studies on HIIT and circuit training providing good examples [2,5,13], or with the use of session-based RPE [18] for the proper manipulation of training loads.

Nevertheless, HIFT has shown the potential to be a safe and effective strategy that could lead to improved performance in untrained and recreationally-trained individuals. Thus, it is possible that HIFT could be a time-efficient training strategy for recreational athletes, as this group would have a greater room for improvement in the characteristics that would lead to sport performance. While HIFT has shown the potential to elicit high levels of mechanical and metabolic stress, comparable to what is seen in training interventions that were effective for high-level athletes, further evidence is needed to support the role of HIFT for fitness improvements in this group. Future research should focus on the direct intervention of HIFT in different sports, and how these sessions can be integrated as part of a yearly training plan.

## Figures and Tables

**Table 1:** High-intensity functional training (HIFT) overview.

Key Points
Sports performance requires the optimal development of multiple fitness components [1]. While resistance training [2,3,4] and high-intensity interval training [5,6,7] are effective strategies, training time is usually limited and strategies that can concomitantly improve metabolic and musculoskeletal fitness in athletes are necessary.
High intensity functional training can provide a time-efficient strategy to develop aerobic power and anaerobic capacity, while simultaneously increasing participants’ muscular endurance, hypertrophy, strength, and power [16,21,25].
Training sessions can be manipulated to focus on musculoskeletal or metabolic components, and can be planned in different ways, determining the type of stress that will be imposed on athletes and the adaptations that will occur.
While research has established the safety of HIFT, sessions still elicit a significant level of fatigue and thus, enough recovery should be provided within the training program.
Even though HIFT has been shown to elicit a significant metabolic and mechanical stimulus [26,27,28,29] with the potential to lead to concomitant improvements in multiple fitness components [21,24,25], studies to date have focused mostly on untrained and recreationally-trained individuals. Future studies are required in order to assess if these findings are transferrable to recreational or high-level athletes. Still, recent evidence suggests that HIFT could lead to improvements in this population [2].

**Table 2:** Sample training session to develop aerobic power and muscle endurance in cross-country skiers.

Exercises	Sets	Reps	Intensity
Ski Erg	As many rounds as possible in eight minutes	100 m	All-out
Thrusters		8	65% 1RM
Medicine Ball Slams		16	50% 1RM

**Table 4:** Sample training session for the development of muscular strength and power in team sport athletes.

Exercises	Sets	Reps	Notes
1. Power Cleans	4	5	Three-minute rest between sets
2a. Back Squats 2b. Box Jumps	4 4	5 5	Rest 20 s between exercises, and three minutes between sets
3a. Deadlifts	3	6	Performed as a short circuit, with two minutes rest between sets
3b. Bench Press	3	6	
3c. Sprints	3	15 m	

**Table 3:** Sample training session for the development of aerobic power in martial arts and combat sports.

Exercises	Sets	Reps	Notes
Row	1	1 min	Perform three to five rounds, with one to two minutes of rest between rounds
Military Press	1	1 min	
Medicine Ball Slams	1	1 min	
Kettlebell Swings	1	1 min	
Sit Ups	1	1 min	
